# Predictors of retreatment after radiofrequency ablation of benign thyroid nodules: role of pre-treatment growth rate and viable volume

**DOI:** 10.1530/ETJ-25-0254

**Published:** 2026-02-25

**Authors:** Adrien Ben Hamou, Jean-Guillaume Marchand, Sylvain Poiree, Cécile Ghander, Camille Buffet, Gilles Russ

**Affiliations:** ^1^ Thyroid Diseases and Endocrine Tumors Department, Pitié-Salpêtrière Hospital, Paris, France; ^2^ Thyroid Unit, American Hospital of Paris, Paris, France; ^3^ Thyroid Tumors Clinical Research Group No 16, Sorbonne University, Cancer Institute, Paris, France; ^4^ CPI, Paris, France

**Keywords:** radiofrequency ablation, thyroid nodules, retreatment, viable volume, pre-treatment growth rate, predictive factors

## Abstract

**Background:**

Radiofrequency ablation (RFA) has emerged as a minimally invasive and effective alternative to surgery for benign thyroid nodules, providing a significant volume reduction and symptom relief. However, approximately one in five patients experiences regrowth requiring retreatment. This study aimed to identify robust predictive factors – spanning the pre-, peri-, and post-treatment periods – to guide patient selection and follow-up strategies.

**Methods:**

In this retrospective cohort study, 293 patients underwent RFA for benign thyroid nodules at Pitié-Salpêtrière Hospital between 2018 and 2023. Retreatment, defined as a second RFA within five years, was the primary outcome. Twelve clinical and treatment variables were analyzed using univariate and multivariate logistic regression. Predictive performance was assessed with receiver operating characteristic (ROC) curves and corresponding area under the curve (AUC) values.

**Results:**

Sixty-one patients (20.8%) required retreatment. Among twelve parameters from three periods (pre-treatment, peri-treatment, and post-treatment), optimal multivariate models identified six significant ones. The pre-treatment growth rate (AUC = 0.808, OR = 1.505, 95% CI: 1.182–1.952, *P* < 0.001) and six-month viable volume (AUC = 0.886, OR = 1.252, 95% CI: 1.088–1.496, *P* < 0.001) showed the highest predictive accuracy. Optimal thresholds were 1.75 mm/year for pre-treatment growth rate and 4.45 mL for viable volume. Patients with viable volume ≤ 2.1 mL had a <5% risk of retreatment, compared to >70% when viable volume exceeded 18.05 mL. Four predictive abacuses were developed to facilitate clinical application.

**Conclusion:**

Pre-treatment growth rate and six-month viable volume are strong, independent predictors of retreatment after RFA of benign thyroid nodules. Incorporating these parameters into clinical decision-making may enhance patient selection, tailor treatment intensity, and inform follow-up intervals, thereby reducing the risk of retreatment and improving cost-effectiveness.

## Introduction

Radiofrequency ablation (RFA) has emerged as a minimally invasive and effective treatment for benign thyroid nodules, offering a significant volume reduction and symptom relief. Recent studies have demonstrated the safety and efficacy of RFA (1), with favorable outcomes in nodule shrinkage and patient satisfaction (2). However, a subset of patients experiences nodule regrowth, necessitating retreatment. Identifying predictors of such regrowth is crucial for optimizing patient selection and improving long-term outcomes. It has been shown that retreatments are more frequent in young patients, in large nodules, in patients with a lower volume reduction at 1 year, and in cases of low-energy delivery (3). Despite these insights, there remains a need for comprehensive analyses to establish reliable predictive markers for retreatment – especially pre-treatment ones – so as to be able to inform the patient before and after the procedure and to allow for individualized care. This study aims to identify significant pre-, peri-, and post-treatment factors that predict the necessity for retreatment following RFA of benign thyroid nodules.

## Methods

This retrospective cohort study analyzed all consecutive patients who underwent RFA for benign thyroid nodules at Pitié-Salpêtrière Hospital between 2018 and 2023. This study was approved by the local ethics committee (IRB number: CRM-2502-457). Benignity was proved by two consecutive benign results with fine needle aspiration cytology (FNAC) interpreted according to the Bethesda system. Surgery was systematically discussed as an alternative treatment option during the pre-treatment consultation, and all patients included in the study opted for RFA after shared decision-making. The procedure was conducted under ultrasound guidance with a free-hand technique and a trans-isthmic approach to ensure stability and precision. The patient was placed in the supine position with mild neck extension, and local anesthesia was administered. An RF Medical Co. generator was used along with an 18-gauge internally cooled electrode (7 cm length, 10 mm active tip). The moving shot technique (4) was applied to ensure homogeneous energy distribution throughout the nodule, minimizing excessive heat propagation. Patients were divided into two groups based on whether they required retreatment within five years. All patients whose nodules had regrown were submitted to another FNA with a benign result. Of note, there was only one patient (not included in the study) whose nodule had regrown but had a cytology result showing atypia of undetermined significance. The final histological result was a tumor of uncertain potential of malignancy. Clinical, ultrasound, and treatment parameters were recorded at baseline, during the procedure, and at six-month follow-up.

The primary endpoint was the need for retreatment, defined as a second RFA session due to persistent compressive symptoms and/or cosmetic complaints, or nodular regrowth. Symptoms were compared at each follow-up using the method described by Mauri *et al.* (5), as cured, improved, or unchanged. Nodular regrowth was defined as an increase of at least 50% in volume compared to the smallest volume observed during the follow-up after RFA. Post-treatment ultrasound follow-up allowed differentiation between viable volume increase, defined as a progressive enlargement of vascularized, non-ablated tissue within the treated nodule, and regrowth, defined as a ≥50% increase in total nodule volume compared with the smallest volume recorded during the follow-up. A viable volume increase was considered an early imaging marker, whereas regrowth was used as a clinical endpoint for retreatment decision-making. Twelve variables were evaluated and divided into three groups:

- Pre-treatment variables (age, sex, nodular EU-TIRADS score, composition, volume (mL), and pre-treatment growth rate defined as (largest diameter at the time of pre-RFA consultation – largest diameter at the time of diagnosis)/number of years from diagnosis to RFA (in mm per year)).
- Treatment variables (delivered energy per volume (J/mL) and ablation time (min)).
- Post-treatment variables (total remaining volume, volume reduction ratio (%), and viable volume in mL at 6 months post-RFA defined as (viable volume = (total volume – ablated volume))). Ablated volume was identified as the hypoechoic, avascular area with increased stiffness on elastography and absence of vascular signals on superb microvascular imaging (SMI), in accordance with previously published definitions. Viable volume therefore corresponded exclusively to the non-ablated, vascularized tissue and not merely to the geometric residual volume (6, 7). Finally, the ablation ratio was defined as (1 − (viable volume/initial volume)) × 100.

Continuous variables were reported as medians with interquartile ranges (IQRs) and compared using the Mann–Whitney *U* test due to non-normal distribution, as assessed by the Shapiro–Wilk test. Categorical variables were expressed as counts and percentages and compared using the chi-square test or Fisher’s exact test, as appropriate. To identify factors independently associated with retreatment, univariate logistic regression was first performed for each variable. Variables with a *P*-value < 0.10 in univariate analysis were included in the multivariate logistic regression model using stepwise selection based on Akaike information criterion (AIC). The final model retained predictors from the pre-treatment (e.g. pre-treatment growth rate and initial volume), peri-treatment (e.g. delivered energy), and post-treatment (e.g. viable volume) periods. Model performance was assessed using the area under the receiver operating characteristic curve (AUC-ROC). Predictive thresholds for continuous variables were determined using Youden’s J index to optimize sensitivity and specificity. Odds ratios (ORs) with 95% confidence intervals (CIs) were reported for all significant predictors. Multicollinearity among variables was assessed using the variance inflation factor (VIF), and no variable exceeded a VIF of 2. The predictive ability of the final model was further evaluated using calibration plots and the Hosmer–Lemeshow goodness-of-fit test. All analyses were conducted using R software, version 4.3.1 (The R Foundation for Statistical Computing, Austria). Statistical significance was defined as a two-sided *P*-value < 0.05.

## Results

Among the 293 patients who underwent radiofrequency ablation (RFA) for benign thyroid nodules between 2018 and 2023, 61 (20.8%) required retreatment within five years, while the remaining 232 (79.2%) were successfully treated with a single session. In the retreatment group, the main indication was persistent compressive symptoms, reported in 70.5% of cases (43 out of 61), followed by regrowth without recurrence of symptoms in 24.6% (15/61) and unsatisfactory volume reduction in 4.9% (3/61), defined as a residual volume greater than 10 mL and/or a volume reduction ratio below 50%. The median interval between the first and second treatment was 23.9 months (interquartile range (IQR): 13.8–40.5), and the median follow-up duration among non-retreated patients was 43.2 months (IQR: 23.1–61.3) (Table 1).

**Table 1:** Patient characteristics. Patients are divided into those requiring a single treatment (*n* = 232) and those requiring retreatment within five years (*n* = 61). Data are presented as median values with interquartile ranges (IQR).

Parameters/category	Total	Retreated patients	
		Yes	No
Number of patients	293	61	232
Autonomous patient			
No	284 (96.9%)	61 (100%)	223 (96.1%)
Yes	9 (3.1%)	0 (0%)	9 (3.9%)
Surgery			
No	293 (100%)	61 (100%)	232 (100%)
Elapsed time (months) between two treatments (retreated patients)			
*n*	61	61	0
Mean ± SD	27.23 ± 15.34	27.23 ± 15.34	
Median (Q1; Q3)	23.92 (13.8; 40.48)	23.92 (13.8; 40.5)	
Minimum; maximum	5.52; 68.3	5.52; 68.3	
Age (years)			
*n*	293	61	232
Median (Q1; Q3)	46 (38; 54)	46 (39; 52)	47 (38; 55)
Minimum; maximum	23; 83	23; 66	23; 83
Sex			
Female	260 (88.7%)	54 (88.5%)	206 (88.8%)
Male	33 (11.3%)	7 (11.5%)	26 (11.2%)
EU-TIRADS score			
3	246 (84%)	53 (86.9%)	193 (83.2%)
4	47 (16%)	8 (13.1%)	39 (16.8%)
Composition			
<50% solid	15 (5.1%)	8 (13.1%)	7 (3%)
50–90% solid	106 (36.2%)	13 (21.3%)	93 (40.1%)
Solid > 90%	172 (58.7%)	40 (65.6%)	132 (56.9%)
Nodule volume on treatment day (mL)			
*n*	293	61	232
Mean ± SD	20.94 ± 14.37	32.87 ± 17.89	17.8 ± 11.43
Median (Q1; Q3)	17.4 (11.1; 26.5)	27.5 (20.1; 42.8)	15.4 (10.2; 22.0)
Minimum; maximum	1.2; 94	6.6; 94	1.2; 68.9
Time since diagnosis (years)			
*n*	293	61	232
Mean ± SD	6.37 ± 5.55	4.03 ± 3.78	6.99 ± 5.77
Median (Q1; Q3)	5 (2; 9)	3 (1; 5)	5 (3; 10)
Minimum; maximum	0; 28	0; 16	0; 28
Growth rate (mm/year)			
*n*	195	40	155
Mean ± SD	2.11 ± 1.84	3.78 ± 2.03	1.69 ± 1.51
Median (Q1; Q3)	1.6 (0.7; 3)	3.38 (2.08; 5)	1.2 (0.6; 2.3)
Minimum; maximum	0; 8.5	0; 8.5	0; 8
Energy (J/mL)			
*n*	293	61	232
Mean ± SD	2,492.43 ± 1,178.05	1,753.87 ± 974.06	2,686.62 ± 1,151.5
Median (Q1; Q3)	2,381 (1,621; 3,169)	1,647 (981; 2,482)	2,604 (1,921; 3,341)
Minimum; maximum	231; 7,245	231; 4,934	600; 7,245
Ablation duration			
*n*	293	61	232
Mean ± SD	20.81 ± 9.26	23.5 ± 9.81	20.11 ± 9
Median (Q1; Q3)	20.38 (14.29; 27.41)	23.28 (16.0; 30.4)	20 (13.36; 25.65)
Minimum; maximum	1; 50.5	5.15; 48.01	1; 50.5
Post-treatment nodule volume (6 months) (mL)			
*n*	293	61	232
Mean ± SD	8.33 ± 7.93	15.21 ± 10.98	6.52 ± 5.69
Median (Q1; Q3)	6.2 (3.2; 10.5)	10.5 (7.8; 20.4)	4.85 (2.7; 8.2)
Minimum; maximum	0.06; 48.8	1.1; 48.8	0.06; 43.5
Volume reduction rate (%)			
*n*	293	61	232
Mean ± SD	64.35 ± 13.25	55.51 ± 13.1	66.68 ± 12.3
Median (Q1; Q3)	64 (56; 74)	56 (46; 64)	67 (58; 75)
Minimum; maximum	10; 99	10; 83	33; 99
Viable volume (mL)			
*n*	293	61	232
Mean ± SD	4.41 ± 6.24	11.09 ± 8.7	2.66 ± 3.83
Median (Q1; Q3)	2 (0.5; 5.7)	7.7 (5.3; 14.2)	1.5 (0.2; 3.52)
Minimum; maximum	0; 42.3	0.6; 42.3	0; 29.6
Ablation ratio			
*n*	293	61	232
Mean ± SD	0.83 ± 0.16	0.68 ± 0.16	0.87 ± 0.14
Median (Q1; Q3)	0.87 (0.74; 0.95)	0.69 (0.55; 0.78)	0.9 (0.81; 0.98)
Minimum; maximum	0; 1	0.1; 0.98	0; 1
Follow-up time (non-retreated patients) (days)			
*n*	232	0	232
Mean ± SD	1,340.47 ± 652.14		1,340.47 ± 652.14
Median (Q1; Q3)	1,315 (796; 1,867)		1,315 (796; 1,867)
Minimum; maximum	185; 2,866		185; 2,866
Proven regrowth (retreated patients)			
No	41 (67.2%)	41 (67.2%)	
Yes	20 (32.8%)	20 (32.8%)	
Symptoms at 6 months (retreated patients)			
No	12 (19.7%)	12 (19.7%)	
Yes	49 (80.3%)	49 (80.3%)	

Baseline demographic characteristics, such as age and sex, were similar between the two groups. The median age was 46 years (IQR: 39–52) in retreated patients and 47 years (IQR: 38–55) in the single-treatment group (*P* = 0.19). Female patients represented 88.5% of the retreatment group and 88.8% of the control group (*P* = 1.00). There was also no significant difference in EU-TIRADS scores, with 86.9% of retreated nodules classified as EU-TIRADS 3 compared to 83.2% in non-retreated patients (*P* = 0.61).

In contrast, several ultrasound and treatment characteristics significantly differed between the groups. Retreated patients had larger initial nodules, with a median volume of 27.5 mL (IQR: 20.1–42.8), whereas the median in the single-treatment group was 15.4 mL (IQR: 10.2–22.0), a statistically significant difference (*P* < 0.001). The pre-treatment growth rate could be assessed only in patients with at least two ultrasound examinations prior to RFA, which was the case for 195 out of 293 patients (66.6%). The median pre-treatment growth rate was also markedly higher among retreated patients, at 3.38 mm/year (IQR: 2.1–5.0), compared to 1.2 mm/year (IQR: 0.6–2.3) in the non-retreated group (*P* < 0.001), suggesting a more aggressive benign behavior in nodules requiring retreatment.

Regarding treatment variables, the median energy delivered per volume was substantially lower in retreated patients at 1,647 J/mL (IQR: 981–2,482) versus 2,604 J/mL (IQR: 1,921–3,341) in patients successfully treated with a single session (*P* < 0.001). In contrast, ablation times were longer in the retreatment group, with a median duration of 23.3 min (IQR: 16.0–30.4) compared to 20.0 min (IQR: 13.4–25.7) in the control group (*P* = 0.017), likely reflecting treatment adjustments in response to larger or more complex nodules (Table 2).

**Table 2:** Univariate analysis of retreatment predictors. Baseline, treatment, and post-treatment characteristics of patients who underwent radiofrequency ablation (RFA) for benign thyroid nodules. Patients are divided into those requiring a single treatment (*n* = 232) and those requiring retreatment within five years (*n* = 61). Data are presented as median (IQR). Pre-treatment growth rate and viable volume at six months post-RFA were the strongest predictors of retreatment, with statistically significant differences (*P* < 0.05). Statistically significant results are presented in bold.

Variable	Single treatment	Retreatment	*P*-value
*n*	232	61	
Age (years)	47 (38; 55)	46 (39; 52)	0.19
Sex (female/male ratio)	206 (88.8%)/26 (11.2%)	54 (88.5%)/7 (11.5%)	1.00
Initial nodule volume (mL)	15.40 (10.17; 22.02)	27.50 (20.10; 42.80)	**<0.001**
EU-TIRADS classification (3/4 ratio)	193 (83.2%)/39 (16.8%)	53 (86.9%)/8 (13.1%)	0.61
Pre-treatment growth rate (mm/year)	1.20 (0.60; 2.30)	3.38 (2.08; 5.00)	**<0.001**
Delivered energy (J/mL)	2,604 (1,921; 3,341)	1,647 (981; 2,482)	**<0.001**
Ablation time (min)	20.00 (13.36; 25.65)	23.28 (16.03; 30.36)	0.017
Total volume at 6 months (mL)	4.85 (2.70; 8.20)	10.50 (7.80; 20.40)	**<0.001**
Volume reduction ratio at 6 months (%)	67 (58; 75)	56 (46; 64)	**<0.001**
Ablation ratio at 6 months (%)	90 (81; 98)	69 (55; 78)	**<0.001**
Viable volume at 6 months (mL)	1.50 (0.20; 3.52)	7.70 (5.30; 14.2)	**<0.001**

Six-month follow-up data also showed significant differences. The median residual nodule volume was 10.5 mL (IQR: 7.8–20.4) in the retreatment group versus 4.85 mL (IQR: 2.7–8.2) in the single-treatment group (*P* < 0.001). The median volume reduction ratio was lower in retreated patients at 56% (IQR: 46–64) compared to 67% (IQR: 58–75) in non-retreated patients (*P* < 0.001). Similarly, viable volume at six months was significantly greater in retreated patients, with a median of 7.7 mL (IQR: 5.3–14.2) compared to 1.5 mL (IQR: 0.2–3.5) in others (*P* < 0.001). The ablation ratio also differed significantly between groups, being lower in the retreatment group (median: 0.69; IQR: 0.55–0.78) than in the single-treatment group (median: 0.90; IQR: 0.81–0.98), *P* < 0.001 (Table 2). No case of new nodule growth outside the treated lesion was observed during the follow-up.

Multivariate logistic regression identified six independent predictors of retreatment: three pre-treatment parameters (pre-treatment growth rate: OR = 1.527; 95% CI: 1.211–1.961; *P* < 0.001, initial nodule volume: OR = 1.058; 95% CI: 1.027–1.096; *P* = 0.001, and composition 50–90% solid vs <50% solid: OR = 0.154; 95% CI: 0.028–0.840; *P* < 0.001), two peri-treatment parameters (delivered energy per volume: OR = 0.875; 95% CI: 0.835–0.911; *P* < 0.001, and ablation time: OR = 1.096; 95% CI: 1.056–1.140; *P* < 0.001), and one post-treatment parameter (viable volume at six months: OR = 1.221; 95% CI: 1.122–1.345; *P* = 0.005). These variables were retained through stepwise selection based on the AIC.

**Figure 1:**
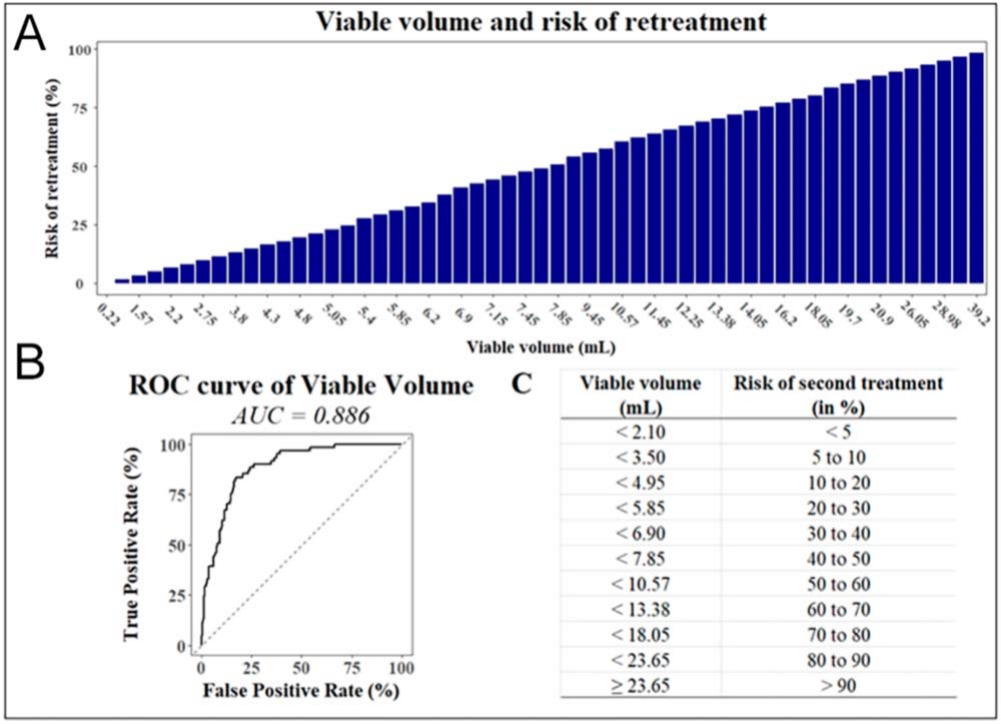
Viable volume and risk of retreatment. (A) Relation between the viable volume and the risk of second treatment. There is a strong relationship between the viable volume measured at six-month follow-up and the risk of a second treatment in the next five years. (B) Receiver operating characteristic (ROC) curves comparing the predictive performance of viable volume at six months (AUC = 0.886) in determining the risk of retreatment. The odds ratio is 1.284 (95% CI: 1.201–1.386, *P* < 0.001), and the optimal cutoff value is 4.45 mL. (C) Abacus used to find the risk of second treatment using the viable volume at six-month follow-up. The abacus is used to inform the patient about the risk of a second treatment. For instance, if the viable volume is 5.85 mL, the risk of retreatment is between 20 and 30%.

**Figure 2:**
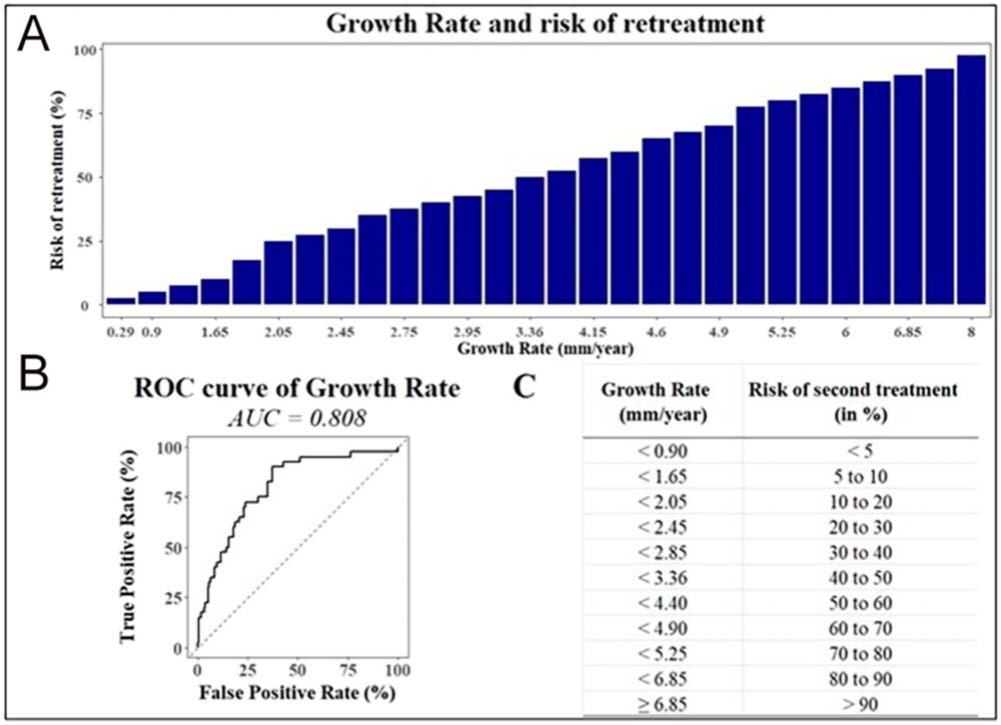
Pre-treatment growth rate and risk of retreatment. (A) Relation between the pre-treatment growth rate and the risk of second treatment. There is a strong relationship between the pre-treatment growth rate measured before RFA and the risk of second treatment in the next five years. (B) Receiver operating characteristic (ROC) curves comparing the predictive performance of pre-treatment growth rate (AUC = 0.808) in determining the risk of retreatment. The odds ratio is 1.835 (95% CI: 1.495–2.306, *P* < 0.001), and the optimal cutoff value is 1.75 mm/year. (C) Abacus used to find the risk of second treatment according to the pre-treatment growth rate parameter. The abacus is used to inform the patient about the risk of a second RFA treatment. For instance, if the pre-treatment growth rate is 2.45 mm/year, the risk of retreatment is between 20 and 30%.

**Figure 3:**
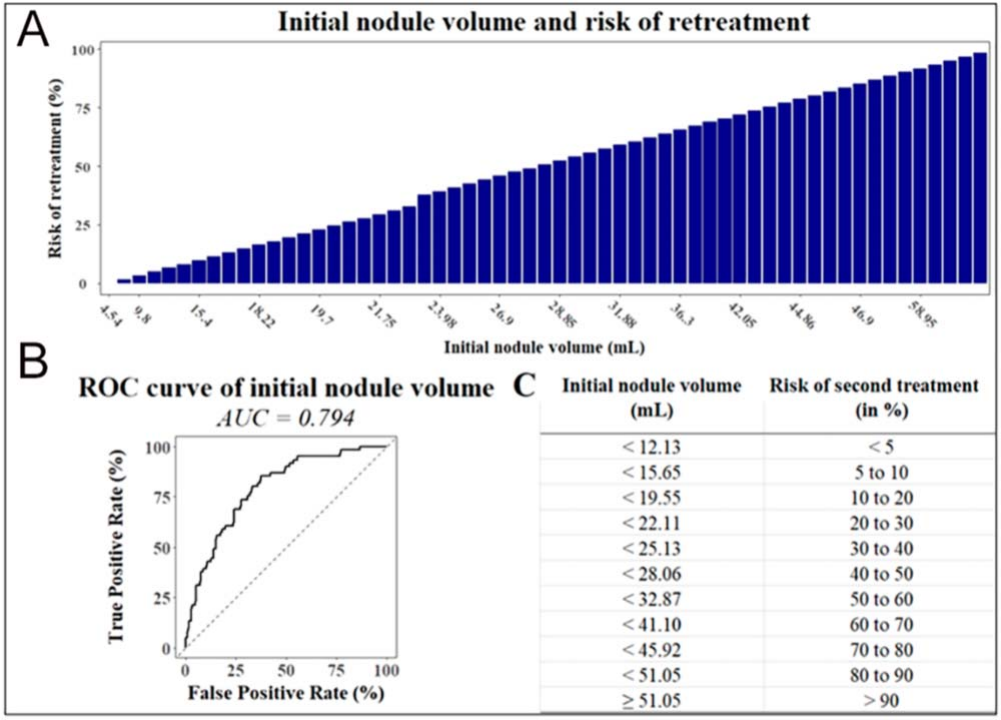
Initial nodule volume and risk of retreatment. (A) Relation between the initial nodule volume and the risk of second treatment. There is a strong relationship between the initial nodule volume and the risk of a second treatment in the next five years. (B) Receiver operating characteristic (ROC) curves comparing the predictive performance of initial nodule volume (AUC = 0.794) in determining the risk of retreatment. The odds ratio is 1.074 (95% CI: 1.051–1.100, *P* < 0.001), and the optimal cutoff value is 18.05 mL. (C) Abacus used to find the risk of second treatment using the initial nodule volume. The abacus is used to inform the patient about the risk of a second treatment. For instance, if the initial nodule volume is 22.11 mL, the risk of retreatment is between 20 and 30%.

**Figure 4:**
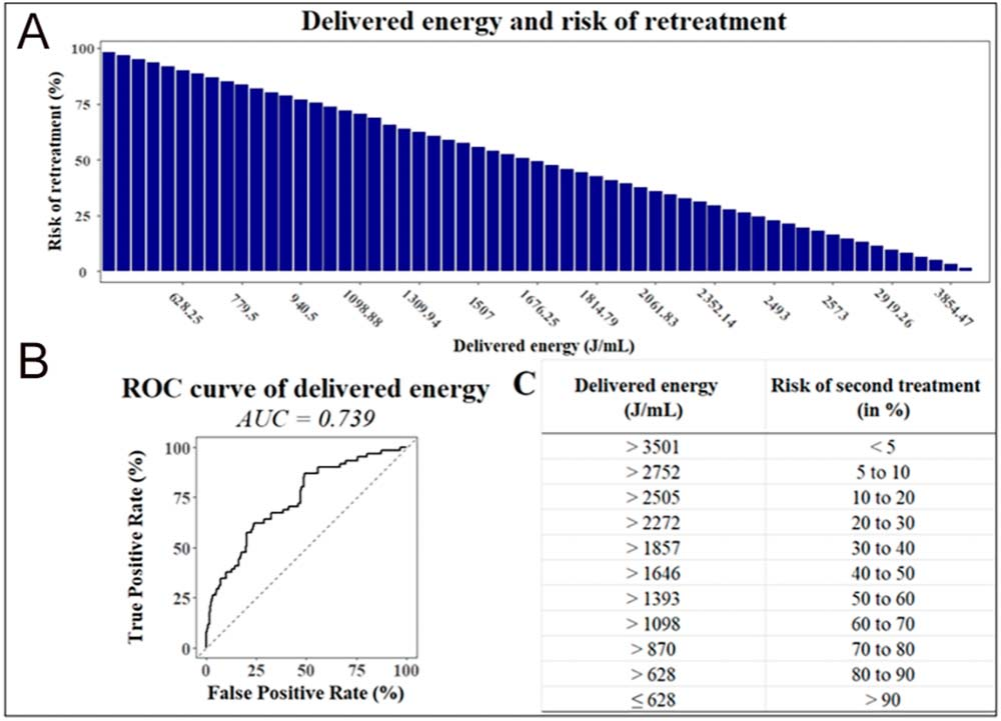
Delivered energy and risk of retreatment. (A) Relation between the delivered energy and the risk of second treatment. There is a strong relationship between the delivered energy and the risk of a second treatment in the next five years. (B) Receiver operating characteristic (ROC) curves comparing the predictive performance of delivered energy (AUC = 0.739) in determining the risk of retreatment. The odds ratio for a 100 J/mL increase is 0.913 (95% CI: 0.881–0.943, *P* < 0.001), and the optimal cutoff value is 1,878 J/mL. (C) Abacus used to find the risk of second treatment using the delivered energy. The abacus is used to inform the patient about the risk of a second treatment. For instance, if the delivered energy is 2,272 J/mL, the risk of retreatment is between 20 and 30%.

Among individual predictors, viable volume at six months showed the highest predictive performance, with an AUC of 0.886 and an optimal cutoff of 4.45 mL (OR = 1.284; 95% CI: 1.201–1.386; *P* < 0.001). Pre-treatment growth rate followed closely, with an AUC of 0.808 and a threshold value of 1.75 mm/year (OR = 1.835; 95% CI: 1.495–2.306; *P* < 0.001). Notably, patients with viable volume ≤ 2.1 mL at six months had a retreatment risk below 5%, whereas those with viable volume exceeding 18.05 mL had a retreatment risk greater than 70%. These findings were visualized through ROC curves and predictive abacuses (Figs 1, 2, 3, 4), offering a practical tool to estimate individual retreatment risk based on measurable clinical parameters.

**Figure 5:**
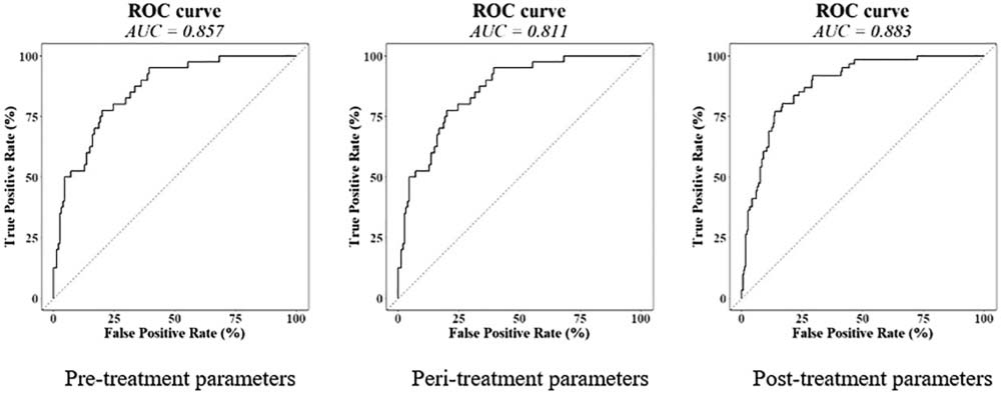
Multivariate analysis of retreatment predictors, considering three periods: pre-, peri-, and post-treatment time. Optimal multivariate logistic regression models identifying significant predictors of retreatment following RFA of benign thyroid nodules (e.g. Table 3). Three multivariate models have been built on parameters of each period (pre-, peri-, and post-treatment), and an algorithm of variable selection (stepwise AIC) has been launched to select the most predictive parameters for each period. Area under the curve (AUC) is provided for the assessment of predictive ability of optimal multivariate models to identify patients at risk of retreatment.

## Discussion

Although RFA has demonstrated its efficacy and safety for treating benign thyroid nodules, the necessity for retreatment remains a concern in a non-negligible proportion of cases. It considerably impacts the total cost of the RFA procedure compared to surgery. Moreover, it can be considered that the complication rate increases linearly at each retreatment. Thus, this study aimed at defining predictors of the risk of retreatment that could be used to select patients with a lower risk and improve RFA technique by optimizing its goals.

To our knowledge, our report is the first one studying as many as twelve variables susceptible to influence the risk of retreatment. It demonstrates that, among these twelve variables, pre-treatment growth rate and viable volume at six months post-RFA are key predictors of retreatment risk.

First, the rate of nodules necessitating a second treatment deserves a comment. In our study, we found that 20.8% patients were retreated. In the multicenter retrospective study by Bernardi *et al.* (3), the rate of retreatment was evaluated in 406 patients treated with either RFA or LA and followed up for 5 years after initial treatment. Twelve percent of patients in the RFA group were retreated. In the retrospective study by Park *et al.* (2), 421 patients with 456 nodules were treated by RFA with a median follow-up period of 90 months. Of these 456 nodules, 190 necessitated multiple sessions, representing 41.6% of all treated nodules. Thus, our result is comparable to the figure reported by Park *et al.* and Bernardi *et al.*

Second, the reasons for retreatment are to be analyzed. In our study, the main reason was the persistence of symptoms (70.5%). This is consistent with the figures reported by Bernardi *et al.* (3), in which 26% of patients (30/115) had symptom relapse and 28% of patients (32/115) underwent a second procedure. In the study by Park *et al.* (2), regrowing nodules were retreated in 33 cases, but the reason why multiple sessions were applied in 157 other nodules was not specified.

Third, we studied which variables could predict the necessity of a second treatment. We found that pre-treatment growth rate and post-treatment viable volume were the strongest predictors.

To our knowledge, this is the first study regarding the interest of pre-treatment growth rate. This was measured as the difference between the longest diameter at the time of RFA minus the longest diameter at the time of diagnosis divided by the time between the two, in mm per year. Faster-growing nodules may indicate a more aggressive benign behavior with rapid cell division. In turn, even a small remaining viable volume after RFA may rapidly expand. A threshold of 1.75 mm/year was identified in our study, suggesting that patients exceeding this rate may rather benefit from alternative treatment strategies, such as surgery. This threshold should not be interpreted as an absolute contraindication to radiofrequency ablation. Rather, a pre-treatment growth rate ≥ 1.75 mm/year should be considered a risk stratification marker, identifying nodules with a higher probability of retreatment. In such cases, alternative treatment options, including surgery, may be discussed, or a more intensive ablation strategy (delivering more energy and targeting a high ablation ratio) and closer post-treatment follow-up may be warranted. Closer monitoring or a repeat ablation session at an earlier stage could also be contemplated.

We also found that the viable volume at six-month follow-up was a strong predictor of retreatment. The cutoff value of 2.10 mL provides a useful clinical marker for identifying patients at risk. Our data suggest that targeting a viable volume <2 mL may significantly reduce the probability of retreatment below 5%, an important threshold for clinical decision-making. Recent studies have emphasized the importance of distinguishing viable volume increase from true nodular regrowth. A viable volume increase reflects early persistence or reactivation of vascularized tissue and may precede macroscopic regrowth by several months or years, as demonstrated by Sim *et al.* and confirmed in a more recent series (8). In our study, viable volume at six months was deliberately used as a predictive marker rather than an outcome, allowing early identification of nodules at a high risk of subsequent regrowth and retreatment. The concept of viable (initially ‘vital’) volume was introduced by Sim *et al.* (7). To evaluate an early sign of regrowth, the authors measured three types of nodule volumes (total volume, ablated volume, and vital volume). Vital volume was defined was defined as the difference between the total remaining volume after treatment and the ablated nodule volume. The latter presents as a hypoechoic area without vascularity with super-resolution Doppler or contrast-enhanced ultrasound. The authors showed that a vital volume increase occurred in 31 nodules (57.4%) and there was regrowth in 13 nodules (24.1%). The mean timing of the vital volume increase was 27.5 ± 18.5 months, and for regrowth, it was 39.9 ± 17.5 months. The vital volume increase tended to precede total nodular volume regrowth by one year. Another indicator has been described by other authors. Yan *et al*. (9) introduced a semi-quantitative index, which was the residual vital ratio (RVR), and found that it was as an independent predictor of regrowth after RFA of symptomatic benign thyroid nodules. The residual vital ratio was calculated as follows: RVR = (viable volume/total volume) × 100, taking into account that viable volume = total volume – ablated volume. Regrowth was observed in 26/206 patients (12.6%). RVR was 56% in the nodules that regrew and 31% in those that did not. In these two reports, the outcome was regrowth and not retreatment. However, in the study by Bernardi *et al.* in 78 patients with 82 benign nodules (10), the authors showed that the RVR was significantly lower in the nodules that did not require any retreatment (31.5 vs 72%). This was consistent with our results, in which the ablation ratio was associated with the risk of retreatment in multivariate analysis.

Unlike these authors, we have chosen to use the absolute value of viable volume and not a ratio like the RVR, on the assumption that it would better predict the risk of retreatment on the one hand and be easier to calculate and use on the other hand. Indeed, the RVR of a nodule with a 20 mL remaining total volume and 2 mL viable volume is the same as the RVR of another nodule with a 2 mL remaining total volume and 0.2 mL viable volume, 10% in both cases. In our study, we have shown that the risk of retreatment is closely related to the absolute value of the viable volume.

The relationship between delivered energy, ablation time, and retreatment risk also merits careful consideration. Bernardi *et al*. (3) reported higher retreatment rates and larger nodules in younger patients, particularly in low-energy delivery cases (with an optimal cutoff of 918 J/mL). In our multivariate logistic regression models (Table 3), only delivered energy per volume (OR = 0.875; 95% CI: 0.835–0.911; *P* < 0.001) and ablation time (OR = 1.096; 95% CI: 1.056–1.140; *P* < 0.001) were confirmed as significant predictors among peri-treatment variables when modeled separately. When integrated into a full model alongside pre- and post-treatment factors, these treatment parameters lost significance in favor of more dominant predictors – pre-treatment growth rate and viable volume at six months (Fig. 5). This suggests that intrinsic nodule characteristics, such as baseline volume and biological behavior (as reflected by pre-treatment growth rate), exert a more profound influence on long-term outcomes than treatment parameters alone. While optimizing energy delivery remains important for achieving adequate initial ablation, our data indicate that treatment adjustments may not fully overcome the underlying risk posed by fast-growing or large-volume nodules.

**Table 3:** Multivariate analysis of retreatment predictors, considering three periods: pre-, peri-, and post-treatment time. Optimal multivariate logistic regression models identifying significant predictors of retreatment following RFA of benign thyroid nodules. Odds ratios (OR) are presented with 95% confidence intervals (CIs). Statistically significant results are presented in bold.

Retreatment predictors	OR (95% CI)	*P*-value
Pre-treatment parameters		
Initial nodule volume (mL)	1.058 (1.027–1.096)	**0.001**
Echostructure (solid)		
50–90% versus <50%	0.154 (0.028–0.840)	**<0.001**
>90% versus <50%	0.472 (0.103–2.212)	0.33
Growth rate (mm/year)	1.527 (1.211–1.961)	**<0.001**
Peri-treatment parameters		
Delivered energy (J/mL)	0.875 (0.835–0.911)	**<0.001**
Ablation time (min)	1.096 (1.056–1.140)	**<0.001**
Post-treatment parameters		
Viable volume (mL)	1.221 (1.122–1.345)	**<0.001**
Ablation ratio at 6 months (%)	0.088 (0.004–1.773)	0.11

An important limitation of our study is that we did not specifically account for the potential influence of operator experience and learning curve effects on retreatment rates. Molecular analyses were not available in this cohort, as all nodules were classified as benign (Bethesda II) on two consecutive FNACs, and molecular testing is not routinely performed in this context. RFA is a highly technique-dependent procedure that requires precise targeting and controlled energy delivery (11), and previous studies have demonstrated that treatment efficacy improves progressively with experience, particularly during the early learning phase (12). Given that our study spans a five-year period, it is likely that refinements in technique, evolving expertise, and improved patient selection contributed to the observed retreatment rates. Future prospective studies should investigate whether retreatment risk declines as operator experience increases and whether the predictive thresholds identified here remain valid across varying levels of proficiency and institutional expertise.

From a clinical perspective, our findings underscore the value of individualized treatment planning. Patients presenting with larger, rapidly growing nodules or with an elevated viable volume at six-month follow-up represent a subgroup at an increased risk of retreatment and may benefit from earlier re-evaluation and consideration of additional RFA sessions before symptomatic recurrence develops (13, 14, 15). Second RFA sessions were effective in controlling symptoms and nodule volume, and no major complications were observed in retreated patients. Integration of these predictive factors into a practical risk stratification model – such as the abacus tool we propose – can aid clinicians in quantifying retreatment risk and tailoring follow-up protocols, thereby enhancing patient counseling and improving long-term outcomes. Based on our findings, a pragmatic follow-up framework may be proposed according to the viable volume measured at six months after the initial RFA. Nodules with a viable volume ≤ 2 mL appear to have a very low risk of retreatment and may therefore undergo standard follow-up. A viable volume between 2 and 4.5 mL identifies an intermediate-risk group that may benefit from closer ultrasound monitoring. In contrast, a viable volume ≥ 4.5 mL is associated with a substantially increased risk of retreatment and may justify early consideration of an additional RFA session, particularly in symptomatic patients.

## Conclusion

Pre-treatment growth rate and six-month viable volume are independent and robust predictors of retreatment after RFA for benign thyroid nodules. Their integration into clinical practice may improve patient selection, treatment planning, and follow-up strategies. Future prospective studies are warranted to validate these thresholds and incorporate them into predictive tools to personalize care and minimize unnecessary re-interventions.

## Declaration of interest

The authors declare that there is no conflict of interest that could be perceived as prejudicing the impartiality of the work reported.

## Funding

This work did not receive any specific grant from any funding agency in the public, commercial, or not-for-profit sector.
